# Economic impact of improving patient safety using Sugammadex for routine reversal of neuromuscular blockade in Spain

**DOI:** 10.1186/s12871-021-01248-2

**Published:** 2021-02-16

**Authors:** J. Martinez-Ubieto, C. Aragón- Benedí, J. de Pedro, L. Cea-Calvo, A. Morell, Y. Jiang, S. Cedillo, P. Ramírez-Boix, A. M. Pascual-Bellosta

**Affiliations:** 1 Hospital Universitario Miguel Servet, Zaragoza, Spain; 2 Hospital Universitario de Móstoles, Madrid, Spain; 3 MSD, Madrid, Spain; 4 Hospital Universitario de la Princesa, Madrid, Spain; 5 MSD Ltd., Hoddesdon, Hertfordshire UK; 6 Covance Clinical Development, Madrid, Spain

**Keywords:** Neuromuscular blocking agents, Sugammadex, Economic impact, Spain, Safety, Surgical procedures, Reversal agents

## Abstract

**Background:**

Neuromuscular blocking (NMB) agents are often administered to facilitate tracheal intubation and prevent patient movement during surgical procedures requiring the use of general anesthetics. Incomplete reversal of NMB, can lead to residual NMB, which can increase the risk of post-operative pulmonary complications. Sugammadex is indicated to reverse neuromuscular blockade induced by rocuronium or vecuronium in adults. The aim of this study is to estimate the clinical and economic impact of introducing sugammadex to routine reversal of neuromuscular blockade (NMB) with rocuronium in Spain.

**Methods:**

A decision analytic model was constructed reflecting a set of procedures using rocuronium that resulted in moderate or deep NMB at the end of the procedure. Two scenarios were considered for 537,931 procedures using NMB agents in Spain in 2015: a scenario without sugammadex versus a scenario with sugammadex. Comparators included neostigmine (plus glycopyrrolate) and no reversal agent. The total costs for the healthcare system were estimated from the net of costs of reversal agents and overall cost offsets via reduction in postoperative pneumonias and atelectasis for which incidence rates were based on a Spanish real-world evidence (RWE) study. The model time horizon was assumed to be one year. Costs were expressed in 2019 euros (€) and estimated from the perspective of a healthcare system. One-way sensitivity analysis was carried out by varying each parameter included in the model within a range of +/− 50%.

**Results:**

The estimated budget impact of the introduction of sugammadex to the routine reversal of neuromuscular blockade in Spanish hospitals was a net saving of €57.1 million annually. An increase in drug acquisition costs was offset by savings in post-operative pulmonary events, including 4806 post-operative pneumonias and 13,996 cases of atelectasis. The total cost of complications avoided was €70.4 million. All parameters included in the model were tested in sensitivity analysis and were favorable to the scenario with sugammadex.

**Conclusions:**

This economic analysis shows that sugammadex can potentially lead to cost savings for the reversal of rocuronium-induced moderate or profound NMB compared to no reversal and reversal with neostigmine in the Spanish health care setting. The economic model was based on data obtained from Spain and from assumptions from clinical practice and may not be valid for other countries.

## Background

Neuromuscular blocking (NMB) agents are administered routinely during surgical procedures to provide muscle relaxation, facilitate the insertion of an endotracheal tube, and prevent patient movement during surgical procedures requiring use of general anesthetics [1].

When neuromuscular blockade is no longer needed to be maintained, patients may either be allowed to spontaneously recover neuromuscular function or be administered a pharmacological reversal agent for more rapid recovery. Spontaneous reversal is neither rapid nor of predictable duration, so frequently, patients may be inadvertently extubated while still experiencing residual neuromuscular blockage [2].

The acetylcholinesterase inhibitor neostigmine is commonly used for reversal of moderate neuromuscular blockade when at least the second twitch (T2) of a train-of-four (ToF) stimulation is present. Recovery of neuromuscular function using neostigmine is also not rapid and its duration may not be predictable [3] which can lead to an extubation of patients while they are still experiencing residual neuromuscular blockage, and, in consequence, the risk of post-operative pulmonary complications including hypoxemia, difficulty breathing and swallowing, upper airway problems, hypercapnia, slurred speech, blurred vision and impaired clinical recovery after surgery [2–10].

This increase in postoperative morbidity can lead to increased length of stay in the post-anesthetic recovery units (PACU), an increased hospital length of stay, and, an increase in the needs and costs of health services [4, 11, 12].

In the past years, new pharmacological alternatives for reversal of neuromuscular blockade have been introduced. Sugammadex (Bridion®, Merck & Co., Inc., Kenilworth, NJ, USA) a modified gamma-cyclodextrin, is a reversal agent available in Spain since 2009 and indicated to reverse neuromuscular blockade induced by the NMB agents rocuronium or vecuronium in adults [13].

In clinical trials, sugammadex has been shown to produce much more rapid and predictable reversal of neuromuscular block compared to neostigmine, in the absence of anti-muscarinic side effects and, in trials where quantitative neuromuscular monitoring was not required, a steep reduction in the incidence of residual NMB [11, 14–17].

In recent years, the number of national and international studies that highlight the increase in complications associated with residual NMB has increased. The frequency of residual NMB ranges between 24 and 32% according to the most recent series, although it has been generally estimated between 6 and 80% depending on the scope of the evaluation, placing it as the main complication in patients undergoing general anesthesia [5, 12, 18–24].

The RECITE-US study estimated the burden and associated risk factors of residual NMB during routine U.S. hospital care. The results of this prospective study showed that 64.7% of the patients had residual NMB (TOF ratio < 0.9%) despite neostigmine administration [25].

At national level, there are several observational studies that evaluate the incidence of residual NMB in Spain [26, 27]. A prospective multicenter study conducted in 26 Spanish hospitals found that 26,7% of a general surgical population in Spain showed residual NMB in the Postanesthesia Care Unit (PACU). Patient-related and procedure-related factors such as female gender, longer duration of surgery, use of benzyl-isoquinolines or halogenated anesthesic use, lack of intraoperative neuromuscular monitoring, and use of neostigmine as reversal agent or no pharmacological reversal were more prevalent in patients showing residual NMB in the immediate postoperative period [26]. Martinez-Ubieto et al. conducted a prospective observational study of cohorts to evaluate the incidence of Postoperative Residual Curarization (PORC) in the PACU and it is relation to the type of muscle relaxant and reversal agent used in 558 patients operated under general anesthesia. In this study, the incidence or residual NMB was much lower when the NMB and reversal agent administered were rocuronium/sugammadex (1.15%) than when it was cisatracurium/neostigmine (28.6%) or when no reversal agent was used (34%) [27].

Currently, reversal of NMB continues to be a safety issue, and so far, the studies related to reversal of NMB at a national level in Spain have focused on pathophysiological, clinical and epidemiological aspects. The aim of this analysis is to estimate the economic impact of introducing sugammadex for routine reversal of rocuronium-induced neuromuscular blockade in Spain.

## Methods

### Model overview

Methodologically, a budget impact analysis makes it possible to evaluate anticipated expenditures for healthcare systems planning to adopt new interventions or introduce changes to the current clinical practice [28]. We developed a budget impact model that projected the aggregated annual net economic impact of using sugammadex instead of neostigmine or no reversal agent in a proportion of procedures were rocuronium is administered. The model was constructed in Microsoft® Excel® 2016 (Microsoft Corp., Redmond, WA, USA).

We collected data from international literature and from official Spanish healthcare databases. Where the available information was insufficient, or data from Spanish sources were considered inaccurate, estimations were provided by an expert panel of Spanish researchers.

The budget impact analysis has been developed in accordance with the Principles of Good Practice for Budget Impact Analysis of the International Society for Pharmacoeconomics and Outcomes Research (ISPOR) [28].

### Model description

This budget impact is based on a comparison between two hypothetical scenarios:

- The current scenario: which represents a situation where sugammadex is not available for routine reversal of NMB
- The alternative scenario: where sugammadex is included as a reversal agent in surgical procedures with moderate and deep NMB.

### Time horizon

The budget impact model projected the economic impact of the introduction of sugammadex in Spain over a one-year time horizon.

### Perspective

The perspective used in this analysis is the Spanish National Healthcare System which only consider direct costs. Pharmacological cost of rocuronium and the reversal agents (neostigmine/atropine and sugammadex) and direct costs of the clinical outcomes were included in the model.

### Procedures

Fifteen types of surgical procedures in which using NMB agents is more frequent were included in the model: appendectomies, hernia repairs, cholecystectomies, colorectal resections, gastric surgeries, intracranial surgeries, spinal cord surgeries, femur surgeries, hip fracture repairs, knee fracture repairs, bronchoscopies or laryngoscopies, vocal cord surgeries, thyroid gland surgeries, prostatectomies, hysterectomies and oophorectomies. Local data from nearly 93% of Spanish public and private hospitals estimated that the annual number of these procedures in 2015 was 733,876 [29] (Table 1). According to Olesnicky et al. [30] cohort study, 73.3% of surgical procedures would use a NMB agent during the surgical procedure.

**Table 1:** Number of surgical procedures carried out in Spain (2015)

Type of	Number of surgical procedures
Appendectomies	44,593
Hernia Repairs	70,618
Cholecystectomies	72,483
Colorectal resections	82,435
Gastric surgeries	123,401
Intracraneal surgeries	20,411
Spinal cord surgeries	49,069
Femur surgeries	21,415
Hip fracture repairs	42,120
Knee fracture repairs	52,493
Bronchoscopies/Laryngoscopies	21,599
Vocal cord surgeries	25,673
Thyroid gland surgeries	21,945
Prostactetomies	31,332
Histerectomies and oophorectomies	54,289
Total anual number	733,876

**Fig. 1:**
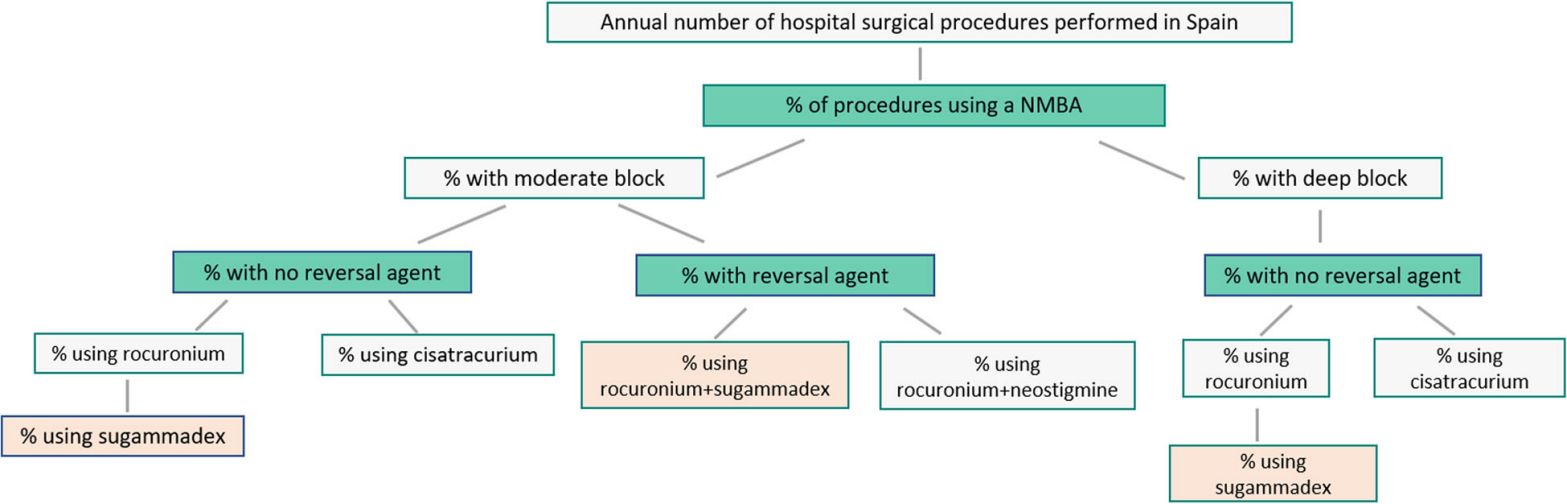
Model structure

The model differentiated between moderate NMB (in our analysis defined as TOF: 1 to 2 twitches) and deep NMB (defined as TOF = 0 and post-tetanic count of 1 to 2). We assumed a split of 80% procedures with moderate NMB and 20% procedures with deep NMB. As there was no literature available from Spain, this assumption was made based on internal market research data, that was validated by experts according to their hospital clinical practice. (Fig. 1).

### Reversal agents

Rocuronium and cisatracurium are the neuromuscular blocking agents included in the model, as vecuronium is not available in our country, representing more than 90% of the market. However, sugammadex is not indicated to reverse cisatracurium-induced NMB [13]. Therefore, the model considered a proportion of instances of cisatracurium use which can be switched to rocuronium, permitting the introduction of sugammadex. It was assumed that 50% of instances in which cisatracurium was used were switched to rocuronium in the alternative scenario using sugammadex as reversal agent. According to the prospective observational study by Martinez-Ubieto et al. [27], rocuronium was administered in 64% of those procedures (Table 2). Due to lack of local data differentiating between moderate and deep NMB, it was assumed the same agent distribution for both. In the cases where neostigmine was considered for reversal it would be co-administered with the anti-muscarinic agent atropine, so the model reflected the co-administration of these two agents.

**Table 2:** Model clinical parameters values: base case

Parameter	Value			
Annual number of hospital surgical procedures	733,876			
Procedures utilizing an NMBA	73.3%			
Type of block	Moderate: 80%		Deep: 20%	
Reversal agent use (neostigmine)	No reversal agent:	Reversal agent:	No reversal agent:	Reversal agent:
	68.0%	32.0%	68.0%	32.0%
Instances with use of rocuronium	64.0%	43.5%	64.0%	43.5%
Instances with use of cisatracurium	25.0%	25.0%	25.0%	25.0%
Instances with use of other agents	11.0%	31.5%	11.0%	31.5%
Proportion of instances where cisatracurium switched to rocuronium	50.0%	50.0%	50.0%	50.0%
	Neostigmine / No reversal agent		Sugammadex	
Risk of post-operative atelectasis	7.3%		1.1%	
Risk of post-operative pneumonia	4.0%		1.9%	

### Clinical parameters

The use of sugammadex and neostigmine has been associated with a risk reduction of two pulmonary complications (atelectasis and post-operative pneumonias). According to the data available when the analysis was performed, both complications were considered as post-operative events of interest for the present analysis [27, 31]. In the case of the risk of post-operative pneumonia, the data combined two sources of information: the Spanish observational studies [27, 31] and a clinical trial [25]. The same risk of post-operative complications was assumed for both for moderate and deep NMB; this point was validated by the expert panel.

Proportion of risk of post-operative events with sugammadex or neostigmine/no reversal are presented in Table 2.

### Costs

List prices of NMB and reversal agents were obtained from Botplus (2019) [32]. A 7.5% discount was applied to drug costs according to Spanish law [33]. Drug costs for specific combinations of NMB agents, reversal agents and block depths are summarized in Table 2. To standardize the cost per dose per patient between different NMB and reversal agents, an average patient weight of 75 kg was assumed. The dose of sugammadex used for NMB reversal was 4 mg/kg for deep NMB and 2 mg/kg for moderate NMB according to the phase III clinical trials described in the sugammadex label [13]. Vial wastage of any unused amount was not considered.

Costs for post-operative atelectasis were derived from the cost per diagnosis-related group (DRG) provided by the Spanish Ministry of Health official statistical site [29]. In the case of post-operative pneumonia, the cost per event was retrieved from regional tariffs extracted from eSalud database [34] (Table 3). Cost of post-operative complications were inflated to € 2019. Following the International Society for Pharmacoeconomics and Outcomes Research (ISPOR) Principles of Good Practice for budget impact analysis costs were not discounted due to the short time horizon used.

**Table 3:** Model economic parameters: base case

Pharmacological costs			
Agent	Dose	Drug Cost	Source
Sugammadex (moderate block)^a^	2 mg	€51.34	BotPlus
Sugammadex (deep block)^a^	4 mg	€102.68	BotPlus
Neostigmine/Atropine	0.5 mg/2 mg	€0.46	BotPlus
Rocuronium	0.6 mg	€1.80	BotPlus
**Post-operative event**	**Cost**		**Source**
Atelectasis	€4999.40		DRG cost. MoH
Pneumonia	€4449.72		eSalud. Oblikue

^a^An average patient weight of 75 kg was assumed in estimating the cost per dose, with vial wastage of any unused amount

**Fig. 2:**
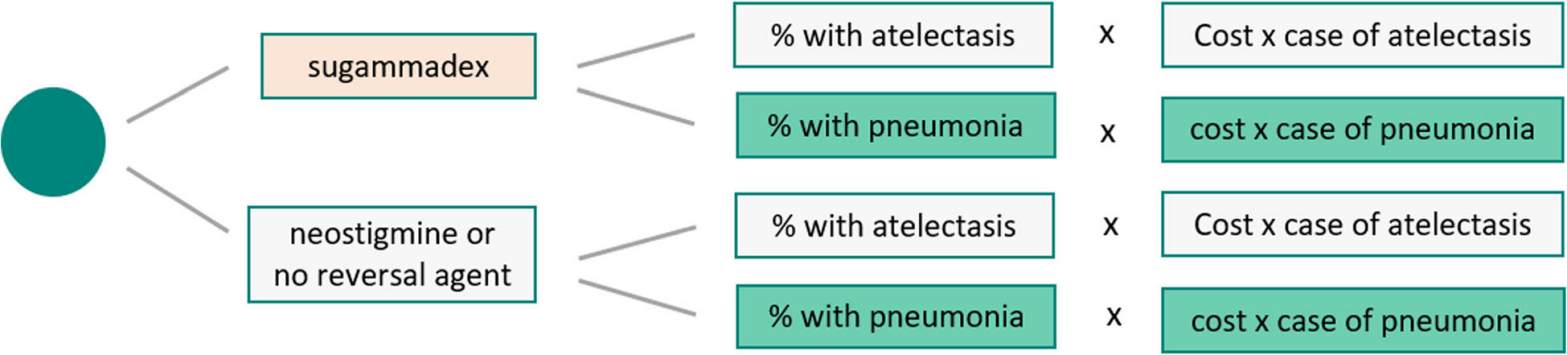
Modeling of clinical cost offsets associated with sugammadex Use

Cost offsets obtained from reducing post-operative pulmonary events with sugammadex compared with neostigmine or no pharmacological reversal were calculated by multiplying the expected number of events (with/without sugammadex) by the cost of each event and calculating the difference between the two scenarios (Fig. 2).

### Sensitivity analysis

Deterministic sensitivity analysis (DSA) was carried out by varying the following parameters included in the model within a range of +/− 50%: proportion of procedures using an NMB agent, risk of post-operative events, cost per event, proportion of cases when sugammadex is used in moderate block and proportion of instances where cisatracurium is switched to rocuronium, proportion of instances rocuronium is used given each reversal agent.

### Model outputs

The results of the analysis are presented in two levels:

1. at procedure level: budget impact of utilizing sugammadex for a specific patient versus neostigmine or non-pharmacological reversal for a single operating room procedure
2. at national level: aggregate budget impact of using sugammadex versus neostigmine or no pharmacological reversal for all procedures for whom sugammadex could be used at national level.

## Results

At procedure level, the introduction of sugammadex is projected to result into a net saving of €249.82 per procedure where this agent could be used (31.2% of the total number of procedures included). Considering the total annual number of hospital surgical procedures, regardless of sugammadex usage, the average net saving per procedure projected was €73.88 (Table 4).

**Table 4:** Procedure-level net budget impact

Block Depth At Reversal	Prior NMBA & Reversal Agent	Prior Scenario Drug Costs	Alternative Scenario (incl. sugammadex) Drug Costs	Total Cost Offsets From Clinical Events Avoided	Net Cost Savings
Moderate	Rocuronium + No reversal	€1.80	€53.14	€307.61	-€256.27
Moderate	Rocuronium + Neostigmine	€2.27	€53.14	€307.61	-€256.73
Deep	Rocuronium + No reversal	€1.80	€104.48	€307.61	-€204.93
	Average per procedure using Sugammadex	€1.97	€59.76	€307.61	-€249.82
	**Average across all surgical procedures** (regardless of sugammadex usage)	€**0.58**	€**17.67**	€**90.97**	**-**€**73.88**

At national level, from the total of 733,876 target procedures considered, sugammadex was used in 228,863 of them (147,542 with rocuronium and no reversal, and 81,321 rocuronium and neostigmine in the prior scenario). The estimated budget impact of the routine introduction of sugammadex in Spanish hospitals was projected to a net saving of €57.1 million annually (Table 5). An increase in drug acquisition costs was offset by savings in post-operative pulmonary events including 4806 post-operative pneumonias (reduction of 52% compared to prior scenario) and 13,996 cases of atelectasis (reduction of 84% compared to prior scenario). The total cost of complications avoided was projected to €70.4 million) (Table 6).

**Table 5:** Annual country-level net budget impact

Block Depth At Reversal	Prior NMBA & Reversal Agent	Prior Scenario Drug Costs	Alternative Scenario (incl. sugammadex) Drug Costs	Total Cost Offsets From Clinical Events Avoided	Net Cost Savings
Moderate	Rocuronium + No reversal	€212,903	€6,272,453	€36,308,198	-€30,248,647
Moderate	Rocuronium + Neostigmine	€184,369	€4,321,505	€25,015,106	-€20,877,969
Deep	Rocuronium + No reversal	€53,225	€3,083,001	€9,077,049	-€6,047,274
	**Total Budget Impact**	€**450,498**	€**13,676,960**	€**70,400,354**	**-**€**57,173,892**

**Table 6:** Number of post-operative events and costs avoided (national-level results)

Block Depth At Reversal	Prior NMBA & Reversal Agent	Number of Post-operative events (Prior Scenario)	Number of Post-operative events (Alternative Scenario)	Costs of post-operative events (Prior scenario)	Costs of post-operative events (Alternative scenario)
Moderate	Rocuronium + No reversal	13,296	3599	€54,335,758	€18,027,560
Moderate	Rocuronium + Neostigmine	9161	2480	€37,435,478	€12,420,372
Deep	Rocuronium + No reversal	3324	900	€13,583,939	€4,506,890
Total		25,781	6979	€105,355,176	€34,954,822
		**4806 pneumonia cases avoided** **13,996 atelectasis cases avoided**		€**70,400,354 costs avoided** **with sugammadex use**	

### Sensitivity analysis

**Fig. 3:**
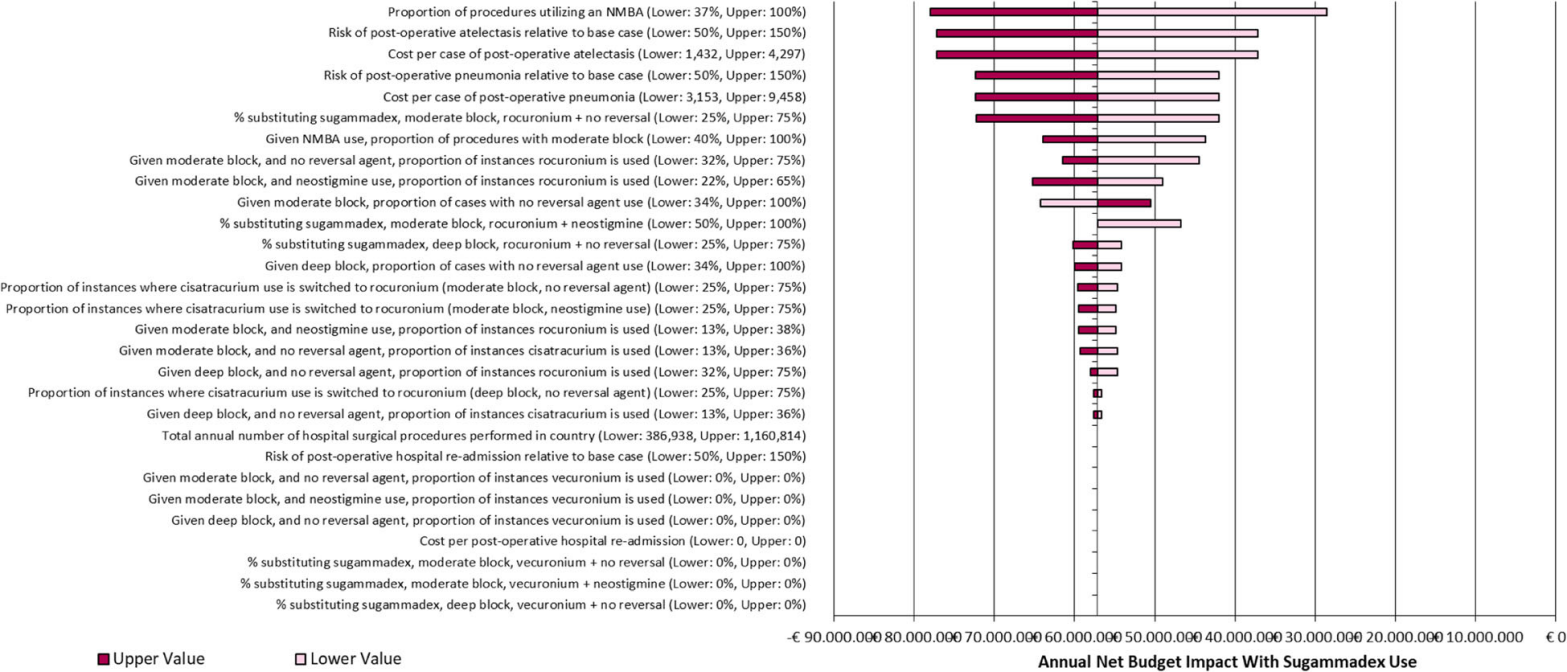
Tornado Diagram. Results from the Deterministic Sensitivity Analysis)

All scenarios tested in the deterministic sensitivity analysis were favorable to the scenario where sugammadex was used (Fig. 3) resulting in cost-saving strategies. According to the sensitivity analysis, the proportion of procedures using a NMB agent and the risk of post-operative atelectasis or pneumonia were the parameters with the greatest impact on the results of the economic model.

## Discussion

Several studies showed that sugammadex can reduce the risk of residual NMB, enhancing operating room efficiency [26, 27]; however, few studies have assessed the economic impact of the improvement of safety outcomes related to the use of sugammadex. In our knowledge, this is the first study to estimate the economic impact of sugammadex based on Spanish real-world data.

There is still a significant incidence of residual NMB in the immediate postoperative period. As it was previously mentioned, different studies described no pharmacological reversal, or reversal with neostigmine as a procedure-related factor more prevalent in patients with pulmonary complications in PACU [26, 27]. Furthermore, an increase number of atelectasis and pneumonias was found in patients who presented residual NMB in the immediate postoperative period and were reversed with neostigmine or spontaneously [22, 23, 35]. Also, the latest study of Capellini et al. [36] evaluated through ultrasound scan the contraction and diaphragmatic movement of patients reversed with neostigmine versus patients with sugammadex. This study concluded that in patients reversed with neostigmine, complete recovery of basal diaphragm function is poorer compared to patients reversed with sugammadex.

This evidence stays in line with other studies where the incidence of postoperative residual neuromuscular blockade was assessed by TOF and where the incidence of atelectasis was higher (7.61%) when reversed with neostigmine compared to patients who were administered sugammadex (1.15%) [27, 31].

Therefore, it is crucial to understand the importance of routine use of reversal agents to avoid the occurrence of complications associated with the neuromuscular blocking drugs resulting in a cost-effective strategy [37–39].

In 2010, two economic analyses assessed the efficiency of reversal agents. The study carried out by Mandim el al [40] showed that the cost per minute of the reversal with sugammadex ($8.34) was lower compared to neostigmine ($104.86). On the other hand, in the UK, Paton et al. [41] conducted a systematic review of randomized controlled trials of sugammadex compared with neostigmine and an economic assessment of sugammadex for the reversal of moderate or deep NMB was performed. The study concluded that the reduction in recovery time associated with sugammadex versus neostigmine was 23.37 min, which resulted in an economic value of £2.87 per minute.

Ozdemir et al. (2010) [42] presented a study comparing the costs of the pulmonary complications associated with the residual NMB using sugammadex and neostigmine. Costs in the spontaneous recovery group were CZK 126.45, CZK 114.56 in the neostigmine group and CZK 34.93 in the sugammadex group.

All these pharmacoeconomic studies have studied direct costs [38–43], however, other indirect aspects have not been evaluated or taken into account, such as the intangible value of the time saved by staff members, the loss of personal and work time of patients and family members, the impact of cancellation of subsequent surgical procedures, reprogramming of the surgical parts or a possible increase in surgical waiting times.

Although more prospective studies are needed, the results of our analysis are aligned with the previous studies conclusions, showing that sugammadex is cost saving compared to neostigmine.

### Limitations

Our study has several limitations, mainly due to the number of assumptions included in the budget impact model. The first limitation is that the split of moderate/deep muscular blockage to 80/20 was based on an internal market research and not in published data. Unfortunately, we did not find published studies supporting, in clinical practice, this distribution. To reduce bias derived from this assumption, we validated this 80/20 split with the investigators according to clinical practice in their hospitals before taken it to develop the economic model. Nevertheless, we have to take into account that this split is an overall estimation and can differ in hospitals where certain specific procedures are more frequent than others (requiring, for example, a higher percentage of patients with deep NMB) or depending on the clinical practice in each hospital or the characteristics of surgical patients. As this could be a critical parameter, one of the scenarios included in the DSA reduced the proportion of moderate block in the distribution and did not result in significant change in the model.

Second, the assumption of the dose of sugammadex used for NMB reversal was of 4 mg/kg for deep NMB and 2 mg/kg for moderate NMB. This assumption was based on the phase III clinical trials described in the sugammadex label, in which the above-mentioned doses were used. Again, this was validated by the investigators based on their clinical practice, but clinical practice can differ from hospital to hospital and the model could not be valid should doses different to those described in the sugammadex label were used.

Another limitation could be the estimation of the cost of post-operative pneumonias and atelectasis. The data was retrieved from the official bulletin of Consejería de Salud de la Junta de Andalucía del 2016 and updated to € 2019 considering inflation. However, these costs, specially the tariff of pneumonia, could have been underestimated because additional complications associated with pneumonia or atelectasis were not taken into account. Depending on the Spanish region and the severity, the cost of a case of pneumonia with derived complications can approach € 8300 [34].

However, the sensitivity analysis suggests that our conclusions are robust and stable across a range of parameter estimates.

Additionally, the model did not consider different risk levels for each post-operative event depending on the type of surgical procedure.

Finally, several potential areas of sugammadex benefit were not explicitly modeled due to lack of evidence to guide modeling, or because related cost offsets would be small relative to those for post-operative atelectasis, or pneumonias. These areas include: 1) avoidance of residual neuromuscular blockade and common sequelae managed routinely and inexpensively in the operating room or post anesthesia care units [44–46]. Examples of this sequelae could be uncomplicated aspiration, hypoxemia, muscle weakness and upper airway obstruction. 2) Operating room time savings under usual standards of care for neuromuscular monitoring and extubation. 3) Avoidance of adverse events associated with neostigmine that could potentially be prevented with sugammadex usage. 4) Improved patient, surgeon and anesthetist satisfaction.

## Conclusion

This economic analysis shows that sugammadex can potentially lead to cost savings for the reversal of rocuronium-induced moderate or deep NMB compared to reversal with neostigmine or no pharmacological reversal in the Spanish health care setting. The economic model was based on data obtained from Spain and from assumptions from clinical practice and may not be valid for other countries.

## Data Availability

All study data are presented either in the article or in the additional files. Access to non-publicly available databases used in the analysis (eSalud and BotPlus) were obtained by subscription. Number of annual surgical procedures was extracted from Conjunto Minimo Básico de Datos (CMBD), a public database which can be accessed from the Spanish Ministry of Health website (https://pestadistico.inteligenciadegestion.mscbs.es/publicoSNS/S/rae-cmbd). The cost per clinical event was retrieved from the eSalud. This platform is the access portal to the Spanish healthcare costs database set up by Oblikue Consulting. eSalud access platform is available on subscription at www.oblikue.com/bddcostes. List prices of NMB agents and reversal agents were obtained from Botplus (https://botplusweb.portalfarma.com/). Botplus is the official database of the *Consejo General de Colegios Oficiales de Farmacéuticos* and, although some of the product information is public, list prices are only available by subscription. Additional data supporting the findings of this study are available from the corresponding author upon reasonable request.

## Abbreviations

DRG: Diagnosis-related group
DSA: Deterministic sensitivity analysis
NMB: Neuromuscular blockade/blocking
PACU: Post anesthesia care units
residual NMB: Residual neuromuscular blockade
T2: Second twitch
ToF: Train-of-four
ISPOR: International Society for Pharmacoeconomics and Outcomes Research
